# Glycated α-lactalbumin based micelles for quercetin delivery: Physicochemical stability and fate of simulated digestion

**DOI:** 10.1016/j.fochx.2022.100257

**Published:** 2022-02-15

**Authors:** Wanting Yin, Luqing Song, Yanan Huang, Fang Chen, Xiaosong Hu, Lingjun Ma, Junfu Ji

**Affiliations:** a College of Food Science and Nutritional Engineering, National Engineering Research Center for Fruit and Vegetable Processing, China Agricultural University, Key Lab of Fruit and Vegetable Processing, Ministry of Agriculture and Rural Affairs, Beijing 100083, China; b Xinghua Industrial Research Centre for Food Science and Human Health, China Agricultural University, Xinghua 225700, China

**Keywords:** Alpha-lactalbumin, Dextran, Quercetin, Maillard glycation, Controlled release

## Abstract

Glycated protein is a kind of promising material that can improve the bioavailability of bioactive compounds and achieve sustained release under digestion. In this study, the α-lactalbumin (ALA)-dextran conjugates synthesized by Maillard reaction were fabricated to load and protect quercetin. Quercetin-loaded micelles stabilized by the ALA-dextran conjugates 1:4 showed the smallest size (428.57 ± 5.64 nm) with highest encapsulation efficiency (94.38% ± 0.50%) of quercetin. Compared to ALA/dextran mixture complex, the conjugates-based micelles had better pH, ionic strength and photothermal stability. Furthermore, the micelles composed of the conjugates 1:2 and 1:4 showed the best controlled release effect during the simulated digestion, releasing 62.41% and 66.15% of quercetin from the total encapsulated contents, respectively, which was mainly related to the resistance of glycated ALA to the enzymes. The findings indicated that ALA-dextran conjugates could be effectively designed for the ideal delivery system of hydrophobic bioactive compounds in food industry.

## Highlights

• ALA-dextran conjugates were fabricated by Maillard reaction.
• The conjugates had the high encapsulation efficiency on loading quercetin.
• The micelles showed excellent pH, ionic strength and photothermal stability.
• The micelles exhibited sustained release of quercetin by the resistance to enzymes.
• The excellent stability made the conjugates promising materials for oral delivery.

## Introduction

Quercetin is a lipophilic bioflavonoid which is abundantly present in the human diet including kale, onion, apples, black tea, and red grapes (Pérez-Jiménez et al., 2011). There is growing evidence suggesting that quercetin has wide spectrum of biochemical and pharmacological activities, including antidiabetic, cardiovascular, anticancer, neuroprotective, and antibacterial activities, which have been largely attributed to their direct antioxidant activities and anti-inflammatory properties (Ulusoy & Sanlier, 2020). However, bioavailability of quercetin, defined as the percentage of an administered dose that reaches the systemic circulation unchanged, is very low, mostly due to its extensive metabolism in the intestine (about 93% in male Sprague-Dawley rats), which reduces its antioxidant potency (Chen et al., 2005, Lesjak et al., 2018). Its high crystallinity and poor aqueous solubility (1 μg/mL) also affect its absorption, limiting its application in the food and pharmaceutical fields (Wang et al., 2016). Hence, a large number of delivery strategies have been extensively studied to improve the oral bioavailability of quercetin, focusing on lipid-, synthetic polymer- and biopolymer-based delivery systems (Bonechi et al., 2018, Ghosh et al., 2017, Penalva et al., 2017, Talarico et al., 2021). Due to their amphiphilicity, biodegradability, biocompatibility and easiness of preparation, protein-based delivery systems have been widely used for quercetin delivery through hydrophobic interactions and self-assemble into nano- or microcarriers. Nevertheless, protein-based delivery systems are generally sensitive to pH variation, ionic strength increasement, and proteolysis of digestive enzymes, which leads to rapid release of the drug in the upper gastrointestinal tract and cannot keep the concentration of the drug at a constant level within the therapeutic window (Alasvand et al., 2017). Therefore, the modification of protein-based delivery system to achieve oral sustained release is of great research value.

Protein glycation has been considered as a promising strategy to improve the functional properties of natural proteins, which is based on the initial stage of the Maillard reaction taken place between the amino groups in proteins and the carbonyl groups in reducing carbohydrates (Liu, Ma, Gao, & Mcclements, 2017). After glycated with polysaccharide, the formed conjugates possess excellent amphiphilicity to encapsulate lipophilic bioactive compounds such as lutein, resveratrol, curcumin, β-carotene and astaxanthin (Davidov-Pardo et al., 2015, Fu et al., 2022, Kim and Shin, 2021, Xu et al., 2014, Zhang et al., 2021). Furthermore, studies have shown that delivery systems comprised of proteins-polysaccharide conjugates had controlled release effect in simulated gastrointestinal digestion (Zhang, Zhou, et al., 2021). The controlled release properties are mainly attributed to the steric hindrance provided by the grafted polysaccharide molecules to maintain the structure of the delivery system, and its strength is determined by the molecular weight of the polysaccharide and the grafting rate of the conjugates (Zhang, Chen, et al., 2021). However, the relationship between the GD and the resistance of the encapsulation structure to digestion and the rate of drug release needs to be further explored.

In order to explore the effect of glycation on the stability, digestibility and release behavior of quercetin delivery system, α-lactalbumin (ALA) and dextran was selected as the model biopolymers. ALA is a major protein component of the whey fraction of bovine milk, which has a molecular mass of 14.2 kDa. ALA exhibited binding affinity with lipophilic bioactive compounds e.g. kaempferol and capsaicin et al, but the encapsulation efficiency (EE) was quite low (Romano, Lajterer, Shpigelman, & Lesmes, 2021). Moreover, native ALA was very sensitive to the hydrolysis of pepsin, it showed rapid and almost completely degradation under the pepsin treatment (El-Zahar et al., 2005). Therefore, ALA was suitable for investigating the improvement of glycation on encapsulation capacity and digestion resistance. Dextran is a glucose polymer mainly formed by α-1,6-glycosidic linkages with good water solubility, commercially selectable molecular weight and high resistance to mammalian digestion (Gallego-Lobillo, Ferreira-Lazarte, Hernández-Hernández, & Villamiel, 2021). Dextran is widely used for protein glycosylation to obtain conjugates with different grafting degrees (GDs) and good EE for lipophilic bioactive compounds (Fan, Yi, Zhang, Wen, & Zhao, 2017). It was reported that ALA-dextran conjugates based nanocomplex for curcumin exhibited high EE (98.8 ± 1.7%) and physicochemical stability, and significantly enhanced DPPH radical scavenging ability of curcumin (Yi et al., 2016). However, to the best of our knowledge, there is few studies focusing on the relationship between the GD, digestion resistance and the release properties of the delivery system based on dextran conjugated proteins for potential oral delivery application.

In this study, quercetin-loaded micelles based on ALA-dextran conjugates were fabricated for the stability and digestibility investigation. Firstly, ALA covalently bounded with dextran (70 kDa) via Maillard reaction by different GDs, and the obtained conjugates were used to load quercetin. The molecular interaction of ALA-dextran conjugates was then investigated. Furthermore, the physicochemical stability and gastrointestinal digestion fate of the quercetin delivery system were also studied to show the advantages of glycated ALA. It aims to give strong evidence and useful reference for the modification of food proteins when acting as wall materials in delivery system.

## Materials and methods

### Materials

Alpha-lactalbumin (total protein content 97.5%, ALA content 92.9%, total fat content 0.2% and lactose content 0.1%) was purchased from Agropur Dairy Cooperative (Appleton, WI, USA). Dextran (MW 70 kDa) was purchased from Shanghai Macklin Biochemical Co., Ltd (Shanghai, China) and quercetin (at least 99% purity, C1386) was purchased from Shanghai Yuanye Bio-Technology Co., Ltd (Shanghai, China). Porcine pepsin (P7000), porcine pancreatin (P7545) and bile salt (48305) were purchased from Sigma-Aldrich Chemical Company (St. Louis, MO, USA). HPLC-grade ethanol and EZ-Run Rec Protein ladder (BP36021) were purchased from Fisher Scientific (Fair Lawn, NJ). All chemicals used were of analytical grade without further purification. Ultrapure water was used in all experiments.

### Preparation of ALA-dextran conjugates

Dextran (3%, *w/v*) was dissolved in ultrapure water with ALA at different molar ratios (1:1, 1:2, 1:4, 1:8). After full hydration, the solutions were adjusted to pH 7 and lyophilized. The powder was ground through a 120-mesh sieve and incubated at 60 °C and 79% relative humidity in an incubator (BPS100CL, Shanghai bluepard instruments Co., Ltd, Shanghai, China) for 24 h. Native ALA was treated under the same condition and used as control. All samples were then collected and stored at −20 °C for further use. In this article, ALA-dextran conjugates prepared at molar ratio 1:1, 1:2, 1:4 and 1:8 were termed as Mai 1:1, Mai 1:2, Mai 1:4 and Mai 1:8, respectively.

### Characterization of ALA-dextran conjugates

#### Gel electrophoresis

Native ALA and ALA-dextran conjugates were analyzed by sodium dodecyl sulfate polyacrylamide gel electrophoresis (SDS-PAGE) using gradient gel (4–20%, Tris-Gly, BeyoGel™). Samples were dissolved in the buffer (50 mM Tris-HCl, pH 6.8, 2% SDS, 0.1% bromophenol blue, 10% glycerol) containing 100 mM β-mercaptoethanol to attend protein content 1.25 g/L. Each solution was denatured at 95 °C for 5 min and ten microliters was loaded to a designated well for electrophoresis. Two separate gels loaded with same samples were run in electrophoresis at the same time. One of them was stained for protein by Coomassie Blue R-250 and the other one was stained for carbohydrate by periodate-Schiff solution. The protein-stained gel was destained with 10% acetic acid (*v/v*) containing 10% methanol (*v/v*) and was scanned with a Gel Documentation Imaging System (JY04S-3C, Beijing JUNYI Electrophoresis Co., Ltd, Beijing, China).

#### Grafting degree (GD)

The extent of glycation was estimated by measuring the free amino groups in ALA and conjugates using OPA assay. 40 mg OPA dissolved in 1 mL 95% ethanol, and mixed with 25 mL of sodium tetraborate buffer (100 mM, pH 9.5), 2.5 mL of 20% (*w/v*) SDS, and 100 µL of β-mercaptoethanol. The solution was diluted to a final volume of 50 mL with distilled water to obtain the OPA reagent. Aliquots of 100 µL of the samples (3 mg protein per mL) were mixed with 2.7 mL of OPA reagent and added into 96-well plates. A blank sample was prepared with 100 µL of deionized water. After incubating at room temperature for 1 min, the absorbance was detected at 340 nm with a microplate reader (Spark 10 M, Tecan, Männedorf, Switzerland). Standard curve was constructed using 0.1–2 mM l-leucine. All assays were performed at least 3 times.

The GD was determined as follows:

(1) $$GD\left(\%\right)=\frac{C_{0}-C_{1}}{C_{1}}\times 100\%$$

where *C_0_* and *C_1_* is the free amino concentration of native ALA (mmol/L) and glycated samples (mmol/L), respectively.

#### Surface hydrophobicity (H_0_)

H_0_ value was determined by using 1,8-anilinonaphthalene sulfonate (ANS) as the florescence probe. The protein solution was diluted by phosphate buffer (10 mM, pH 7.0) to the concentration ranging from 0.05 to 0.5 mg/mL, and then ANS (40 µL, 8 mM) was added to 4 mL of each diluted sample solution. Fluorescence intensity (FI) was measured at 390 nm (excitation) and 470 nm (emission) using the F-4500 florescence spectrophotometer (Hitachi Ltd, Tokyo, Japan) with a slit width of 5 nm. The initial slope of FI versus protein concentration plot was used as the index of H_0_.

#### Fourier transform infrared (FTIR) spectroscopy

The spectroscopic measurements were performed using 2 mg of each sample mixed with 198 mg of dried KBr. FTIR spectra were measured by a FTIR spectrometer (Tensor 27, Bruker, Karlsruhe, Germany) from 400 to 4000 cm^−1^ with a resolution of 4 cm^−1^ in 64 scans. The recorded spectra were then analyzed by OMNIC 8.0 software (Nicolet Instruments Corporation, USA).

### Preparation of quercetin-loaded micelles

ALA, glycated ALAs and physical mixture of ALA and dextran at ratio of 1:4 were separately dissolved in deionized water at a protein concentration of 1 mg/mL. Then, 0.2% (*w/v*) quercetin ethanol solution was slowly injected into protein solutions by syringes with magnetic stirring. The final ratio of quercetin to protein was 4:5. The ethanol was removed by rotary evaporation (RE-2000A, Yarong, Shanghai, China) operated at 40 °C to form an aqueous dispersion of micelles. After that, the samples were treated by high-pressure homogenization (1400 bar, three times of cycle) by using a high-pressure homogenizer (JN-10HC, Guangzhou Juneng Nano & Bio Technology Ltd, Guangzhou, China). The insoluble unencapsulated quercetin in the samples was removed by centrifugation (8000*g*, 10 min). The supernatants were then lyophilized and stored in a desiccator for further analysis. In this article, quercetin-loaded encapsulation system based on ALA, Mai 1:1, Mai 1:2, Mai 1:4, Mai 1:8 and mixture of ALA and dextran were termed as ALA-Q, Mai 1:1-Q, Mai 1:2-Q, Mai 1:4-Q, Mai 1:8-Q and Mix 1:4-Q, respectively.

### Characterization of quercetin-loaded micelles

#### Determination of particle size and ζ-potential

The particle sizes, polydispersity index (PDI) and ζ-potential of quercetin-loaded encapsulation systems were measured by a particle size analyzer (Zetasizer ZEN 3700, Malvern Instruments Ltd, Worcestershire, UK) at 25 °C. All measurements were carried out at 25 °C and the results were reported as the average of three readings.

#### Encapsulation efficiency (EE) and loading capacity (LC) of quercetin

10 mg of freeze-dried quercetin-loaded sample was mixed with 5 mL of methanol, vortexed for 30 s and centrifuged at 10,000*g* for 10 min to wash away the free quercetin on or near the surfaces of the particles. Supernatants were collected to quantify the free quercetin. Another 10 mg sample was firstly dissolved in 0.5 mL water, then mixed with 4.5 mL methanol, to obtain the total content of quercetin in the sample. The methanol aqueous solution was also centrifuged (10,000*g*, 10 min) to precipitate protein and polysaccharide. Quercetin is quantified using high performance liquid chromatography (HPLC) (Shimadzu corporation, Kyoto, Japan) with C18 column (GL Sciences Inc., 4.6 × 150 mm, 5 μm). The detection wavelength was 370 nm and the injected solution was 20 μL. The mobile phase made up of methanol/water (70:30, *v/v*) was freshly prepared and degassed before use, and the flow rate was set at 1.0 mL/min. The EE and LC values were calculated using the following equations:

(2) $$\text{EE(\%)=}\frac{Total\;amount\;of\;quercetin-free\;quercetin}{\mathrm{Total}\;amount\;of\;quercetin}$$

(3) $$\text{LC(\%) =}\frac{Total\;amount\;of\;quercetin-free\;quercetin}{Total\;weight\;of\;micelles}$$

#### Intrinsic fluorescence emission spectroscopy

The intrinsic fluorescence of native ALA, Mai 1:1, Mai 1:2, Mai 1:4, Mix 1:4 and their corresponding quercetin-loaded encapsulation systems were carried out via the florescence spectrophotometer (F-7000, Hitachi, Tokyo, Japan). The protein concentrations of all samples were adjusted to 28 μg/mL. The excitation wavelength was 280 nm, and the emission spectrum was recorded from 300 nm to 500 nm (both at a constant slit of 10 nm).

#### Transmission electron microscopy (TEM) analysis

The morphological structures of freshly prepared Mai 1:1-Q, Mai 1:2-Q, Mai 1:4-Q and Mix 1:4-Q were observed by TEM (HT-7700 Hitachi, Japan). The dispersion of samples was diluted, and dropped on the carbon-coated copper grid. Excess dispersion was removed by filter paper, and then the copper grid was stained using uranyl acetate for 3 min.

### pH, ionic strength and photochemical stability

All stability studies used newly prepared Mai 1:1-Q, Mai 1:2-Q, Mai 1:4-Q and Mix 1:4-Q. For pH stability, the samples were adjusted to pH 3.0 to 8.0 with 1 M HCl or 1 M NaOH. The salt stability of composite nanoparticles was determined by adding different amounts of NaCl to these dispersions to give final salt concentrations ranging from 0 to 500 mM. The particle size and photographs of the dispersions were recorded after they were exposed to above conditions for 3 h.

For the stability of quercetin after different UV treatment time, aliquots of free quercetin and quercetin-loaded encapsulation systems were diluted to quercetin concentration of 500 μg/mL and 10 mL samples were directly exposed to a UV lamp (365 nm, 35 W) 10 cm away for 0–300 min. The quercetin concentration was measured by HPLC according to the method described above in Section 2.5.2.

### In vitro simulated gastrointestinal digestion

#### Release profiles of quercetin

The *in vitro* digestion process was based on the INFOGEST 2.0 method with some modifications (Brodkorb et al., 2019). The Mai 1:1-Q, Mai 1:2-Q, Mai 1:4-Q and Mix 1:4-Q solutions were prepared to protein concentrations of 0.2 mM. Quercetin dispersed in water was used as a control. For simulated gastric digestion, 10 mL of liquid sample was mixed with 10 mL of Simulated Gastric Fluid (SGF) and adjust pH to 3.0. The mixed solution was incubated at 37 °C for 2 h under continuous shaking (110 rpm) by a water bath shaker (SHZ-C, Shanghai Boxun Medical Bio-instrument Co., Ltd, Shanghai, China). At the end of gastric digestion, the pH of the simulated chyme was adjusted to 7.0 and mixed with 20 mL Simulated Intestinal Fluid (SIF) for another 2 h’ intestinal digestion. Every 30 min, 1 mL of digested samples were collected and immediately frozen by liquid nitrogen. The frozen samples were lyophilized and the obtained powders were mixed with 1 mL of methanol, shortly vortexed and centrifuged (10,000*g*, 10 min). Quercetin in the supernatants were recognized as released part and quantified by HPLC.

#### SDS-PAGE

During digestion, 50 μL of the Mai 1:1-Q, Mai 1:2-Q, Mai 1:4-Q and Mix 1:4-Q digested samples were separately collected from digestive fluids at 5, 30, 60, 90 and 120 min of gastric and intestinal digestion, respectively. The taken samples were immediately frozen by liquid nitrogen and lyophilized, and then dissolved by 40 μL sample buffer. The following operations of electrophoresis and staining were described above in Section 2.3.1.

#### Particle size distribution

The particle size distribution of the digested samples after 30 min and 120 min of digestion in SGF and SIF were measured with a laser particle size analyzer (LS 13 320, Beckman Coulter, Inc., Fullerton, CA, USA). An optical model including polarization intensity differential scattering (PIDS), 41% PIDS obscuration, and 60% pump speed were used.

### Statistical analysis

All measurements were repeated at least three times. Data were recorded as mean ± standard deviation and statistically analyzed for a significant difference (p < 0.05) by IBM SPSS statistics 26 (IBM, NY, USA). All curve fittings were applied by the program Origin 2021b (OriginLab Corporation, MA, USA).

## Results and discussion

### Characterization of ALA-dextran conjugates

#### SDS-PAGE

**Fig. 1:**
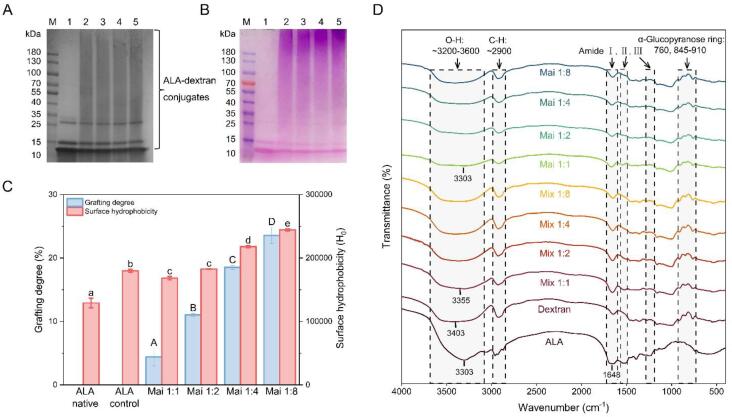
Characterization of ALA–dextran conjugates. (A) Protein stain and (B) carbohydrate stain of SDS-PAGE patterns. Lanes 1–5: native ALA, ALA–dextran conjugate Mai 1:1, Mai 1:2, Mai 1:4, Mai 1:8. (C) The grafting degrees and surface hydrophobicity (p < 0.05). (D) The FTIR spectra of ALA, dextran, ALA/dextran mixtures and ALA-dextran conjugates.

In order to confirm the formation of glycation conjugates, SDS-PAGE was performed on ALA-dextran Maillard products with different molar ratios (ALA: dextran = 1:1, 1:2, 1:4 and 1:8). Under the reducing conditions, only covalently linked conjugates can be remained on the gels. As shown in Fig. 1A (protein stain), the molecular weight corresponding to the native ALA bands were consistent with the electrophoresis results in other studies (lane 1) (Joubran, Moscovici, & Lesmes, 2015). After Maillard reaction, the ALA bands were slightly diminished, and new smeared zones were clearly appeared from the top to the bottom of the gel (lane 2–5). Correspondingly, diffuse bands were also shown in carbohydrate staining gel (lane 2–5, Fig. 1B). Combining the two staining results demonstrated that dextran was successfully attached to ALA, and the heterogeneous reaction between multiple free amino groups in the protein and the reducing end carbonyl groups in the polysaccharide leaded to the broad molecular weight distribution (Zhou et al., 2012). There is no significant difference between the diffuse bands of samples with different ALA - dextran ratios, probably attributed to the insufficient increase of conjugated dextran (Zhou et al., 2012).

#### Grafting degree (GD) and surface hydrophobicity (H_0_)

The OPA assay shows that the GD increased from 4.40% to 22.85% with the rise of ALA-dextran molar ratio from 1:1 to 1:8 (Fig. 1C). Each ALA molecule contains 13 amino groups, including 12 lysine residues and the *N*-terminal residue (Deng, Wierenga, Schols, Sforza, & Gruppen, 2017). This implied that on average, there were 0.6, 1.4, 2.4 and 3.0 dextran molecules were attached to each ALA molecule for Mai 1:1, Mai 1:2, Mai 1:4, Mai 1:8, respectively. Upon dry heating at 60 °C, H_0_ of the ALA control increased significantly compared with the native ALA, suggesting the exposure of more hydrophobic groups. Going through the same heat treatment with dextran, Mai 1:1 exhibited lower H_0_ than ALA control, and as the GD value raised, H_0_ displayed a progressive trend of elevate. Generally, heating leads to some degree of denaturation of a protein, resulting in structure unfolding and migration of the internal hydrophobic groups to the surface. However, the Maillard reaction could either promote or hinder the exposure of hydrophobic groups of the surface of protein. On one hand, covalent binding of dextran to ALA introduced a large number of hydroxyl groups, increased the surface hydrophilicity and shielded the contact between ANS and hydrophobic domains of ALA (Nasrollahzadeh, Varidi, Koocheki, & Hadizadeh, 2017). On the other hand, the attachment of macromolecular polysaccharides might stretch protein, resulting in structural expansion and further exposure of hydrophobic groups (Joubran, Moscovici, Portmann, & Lesmes, 2017). Here, the structural stretching effect of the grafted dextran dominated, causing the H_0_ to increase with the increase of the GD.

#### Fourier-transform infrared spectroscopy (FTIR)

As shown in Fig. 1D, the characteristic bands of ALA included amide I (C—O stretching), amide II (N—H bending), amide III (C—N stretching and N—H deformation) bands at 1700–1600 cm^−1^, 1600–1500 cm^−1^ and 1250–1350 cm^−1^ respectively. The absorption peaks of α-glucopyranose ring deformation modes in the structure of dextran located at 770, and 845–910 cm^−1^ (Jung, 1995). The wide absorption ranged from 3600 to 3200 cm^−1^ and the peak closed to 2900 cm^−1^ existed in both ALA and dextran were related to O—H groups and the antisymmetric stretching of C—H in CH_2_ and CH_3_ groups, separately.

In the case of ALA-dextran mixtures, characteristic absorption peaks of both protein and polysaccharide could be found. After Maillard reaction, ALA-dextran conjugates showed weaker absorptions at amide bands than mixtures. Moreover, with larger proportion of dextran, the intensity at amide II gradually disappeared. These changes were similarly described by previous studies, suggesting the consumption of –NH_2_ groups and the generation of Schiff base (C=N) (Cheng, Mu, Jiao, Xu, & Chen, 2021). The absorptions caused by consecutive α-glucopyranose rings in the structure of dextran were stronger in glycated products than ALA, but weaker than mixtures, which also indicated successful attachment of dextran to ALA (Liu et al., 2021). In addition, the absorption bands of the conjugates in the 3200–3500 cm^−1^ range slightly shifted compared to the mixtures, which may be due to the hydrogen bonding between ALA and dextran (Pirestani, Nasirpour, Keramat, & Desobry, 2017). Furthermore, the hydroxyl content was observed to increase with the proportion of dextran, which might be related to the increase of GD. The analysis above indicated the generation of covalent bonds between ALA and dextran during Maillard reaction, and hydrogen bonds might also be involved.

### Effect of ALA-to-dextran ratio on particle properties, encapsulation efficiency (EE) and loading capacity (LC)

In this study, ALA-dextran conjugates obtained by Maillard reaction were used to load quercetin. The quercetin complexed with native ALA and ALA/dextran mixture at a molar ratio of 1:4 (Mix 1:4-Q) was also fabricated as contrasts. The particle size, PDI value, zeta-potential, EE and LC were summarized in Table 1 and the size distribution was shown in Fig. S1 of Supporting Information. Particle size of quercetin-ALA complex was over 3 μm, and the PDI was 0.704 ± 0.257, indicating that ALA was incapable of forming a stable encapsulation system. As for the micelles based on glycated ALA, Mai 1: 4 had the smallest size at 428.57 ± 5.64 nm, and the diameter of Mai 1:1-Q and Mai 1:2-Q were 516.77 ± 4.44 nm and 443.07 ± 1.93 nm, respectively. The PDI value of these three were all below 0.25, demonstrating the formation of uniform micelles. Interestingly, Mix 1:4-Q also had a relatively small particle size at 510.23 ± 3.87 nm. However, micelles Mai 1:8-Q had a mean size over 1 μm. Compared to quercetin-ALA complex, significantly reduced particle size and improved stability of glycated ALA were mainly attributed by the presence of dextran, which generated a strong steric repulsion to prevent proteins aggregation (Kim & Shin, 2021). The outer layer of dextran also shielded the negative charge of ALA, resulting in a decrease in the absolute values of zeta potential.

**Table 1:** Characteristics of quercetin-loaded micelles.

Carrier material	Size (nm)	PDI^a^	Zeta potential (mV)	EE^b^ (%)	LC^c^ (%)
Native ALA	3728.33 ± 296.52^a^	0.704 ± 0.257^a^	−13.83 ± 0.49^a^	10.44 ± 3.36^e^	1.02 ± 0.73^b^
Mai 1:1	516.77 ± 4.44^b^	0.243 ± 0.015^b^	−6.56 ± 0.26^b^	44.22 ± 2.71^d^	2.14 ± 0.22^a^
Mai 1:2	443.07 ± 1.93^b^	0.187 ± 0.017^b^	−0.74 ± 0.06^e^	70.35 ± 1.81^c^	2.18 ± 0.14^a^
Mai 1:4	428.57 ± 5.64^b^	0.182 ± 0.021^b^	−3.79 ± 0.28^c^	94.38 ± 0.50^a^	2.17 ± 0.02^a^
Mai 1:8	1395.33 ± 57.83^a^	0.348 ± 0.063^b^	−2.69 ± 0.51^d^	89.85 ± 1.67^a^	0.54 ± 0.05^b^
Mix 1:4	510.23 ± 3.87^b^	0.209 ± 0.003^b^	−6.60 ± 0.02^b^	81.61 ± 1.87^b^	1.87 ± 0.09^a^

a Polydispersity index.

b Encapsulation efficiency.

c Drug loading capacity.

It was observed that quercetin-ALA complex possessed low EE and LC values, which were only 10.44 ± 3.36% and 1.02 ± 0.73%. However, when dextran exists, most of samples displayed remarkable increased EE values in ascending order of glycation degree (p < 0.05), especially for Mai 1:2-Q and Mai 1:4-Q, which could reach 70.35 ± 1.81% and 94.38 ± 0.50% for EE, as well as 2.18 ± 0.14% and 2.17 ± 0.02% for LC, respectively. This result was related to the increasing trend of H_0_, in which the exposure of protein hydrophobic part could enhance the ability of glycated protein to bind hydrophobic compounds. As for Mai 1:8-Q, the high GD had hindered the formation of stable micelles and affected the EE and LC values, which were both lower than Mai 1:4-Q. Furthermore, the EE of Mai 1:4-Q was significantly higher than that of Mix 1:4-Q (p < 0.05). Combined with the results of particle size and zeta potential, the best encapsulation effect can be obtained by ALA-dextran conjugates with molar ratios of 1:4.

### Characterization of quercetin-loaded micelles

**Fig. 2:**
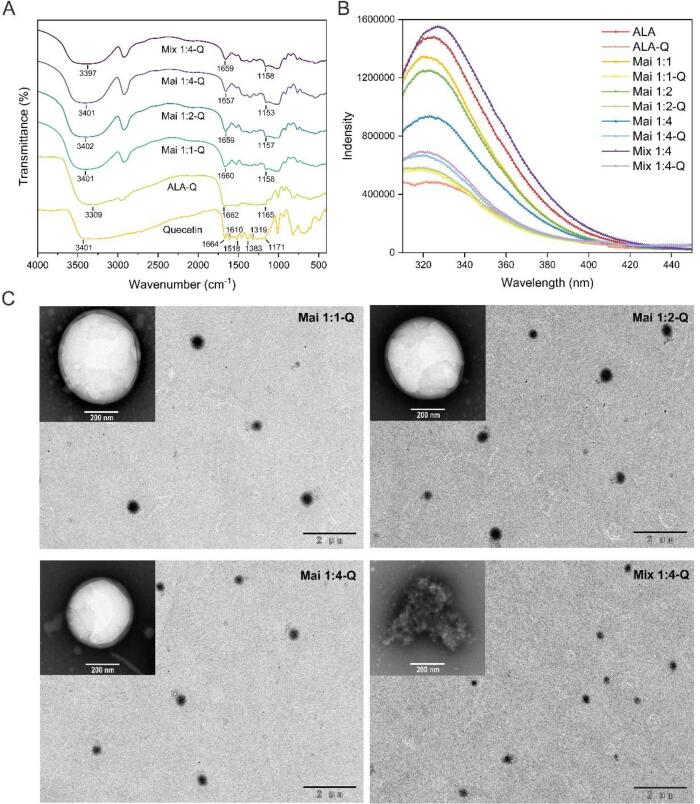
(A) The FTIR spectra of quercetin, ALA-Q, Mai 1:1-Q, Mai 1:2-Q, Mai 1:4-Q, Mix 1: 4-Q. (B) Florescence spectrum of ALA, ALA-Q, Mai 1:1, Mai 1:1-Q, Mai 1:2, Mai 1:2-Q, Mai 1:4, Mai 1:4-Q, Mix 1: 4, Mix 1: 4-Q. (C) TEM images of Mai 1:1-Q, Mai 1:2-Q, Mai 1:4-Q, Mix 1: 4-Q.

Fig. 2A describes the FTIR spectra of quercetin and the micelles. For the spectrum of native quercetin, the characteristic bands were displayed at 3401 cm^−1^ (O—H stretching), 1610–1518 cm^−1^ (C=C aromatic bonds stretching), 1383 cm^−1^ (aromatic ring of the phenolic moiety), 1319 cm^−1^ (=C—O—H phenolic group stretching), and 1171 cm^−1^ (catechol moiety on the aromatic ring stretching) (Porto et al., 2018). Many of these sharp peaks in the 500–1610 cm^−1^ region observed in the quercetin spectrum disappeared in the spectra of Mai 1:1-Q, Mai 1:2-Q and Mai 1:4-Q and Mix 1:4-Q, while only weakened in quercetin-ALA complex. This result indicated that micelles with higher EE had better effect to mask the characteristic peaks of quercetin. In addition, when quercetin was incorporated into the micelles, the peaks in the coat materials assigned with hydroxy stretching vibrations moved from 3303, 3303, 3286, 3306, 3401 cm^−1^ (ALA, Mai 1:1, Mai 1:2, Mai 1:4 and Mix 1:4) to 3309, 3401, 3402, 3401, 3397 cm^−1^ (ALA-Q, Mai 1:1-Q, Mai 1:2-Q, Mai 1:4-Q and Mix 1:4-Q), respectively, confirming the presence of hydrogen bonding between quercetin and the coat materials (Fu et al., 2022). Moreover, the shift of the peaks from 1648, 1666, 1658, 1660 and 1652 cm^−1^ to 1662, 1660, 1659, 1657 and 1659 cm^−1^ (amide I, C—O stretching), and 1083, 1110, 1113, 1111 and 1107 cm^−1^ to 1165, 1158, 1157, 1153 and 1158 cm^−1^ (C—O—C stretching) in the nutraceutical loaded biopolymer micelles were consistent with hydrophobic interactions among the ALA and quercetin (Chen et al., 2020).

In Fig. 2B, tertiary structural changes of ALA and the binding between micelles and quercetin were detected via intrinsic florescence spectroscopy of the tryptophan and tyrosine residues of the protein at excitation wavelength of 280 nm. With the increasing molar ratio of dextran, the florescence intensity of glycated ALA showed a remarkable decline, owing to the rise of graft degree enhanced the shielding effects of conjugated dextran, which blocked the florescence signals of tryptophan and tyrosine residues (Jiménez-Castaño, Villamiel, & López-Fandiño, 2007). In addition, after quercetin was encapsulated, the fluorescence intensity of all the carriers obviously dropped, indicating that the binding of hydrophobic quercetin aromatic rings near the tryptophan residues quenched the fluorescence of the protein (Wu, 2016). This phenomenon indicated the successful binding of small quercetin molecules to ALA in all five encapsulation systems through hydrophobic interactions and hydrogen bonds (Romano, Lajterer, Shpigelman, & Lesmes, 2021).

The TEM images (Fig. 2C) showed that both quercetin-loaded micelles stabilized by ALA-dextran conjugates and physical mixtures had a spherical shape, and most of the micelles had diameters in the range of 400 nm–500 nm, which were consistent with the results obtained using dynamic light scattering measurements (Table 1). However, there were some broken and irregular particles in Mix 1:4-Q possibly due to the weak non-covalent interactions, while micelles based on Maillard productions were almost all intact spheres.

### Stability study of quercetin-loaded micelles

#### pH stability

**Fig. 3:**
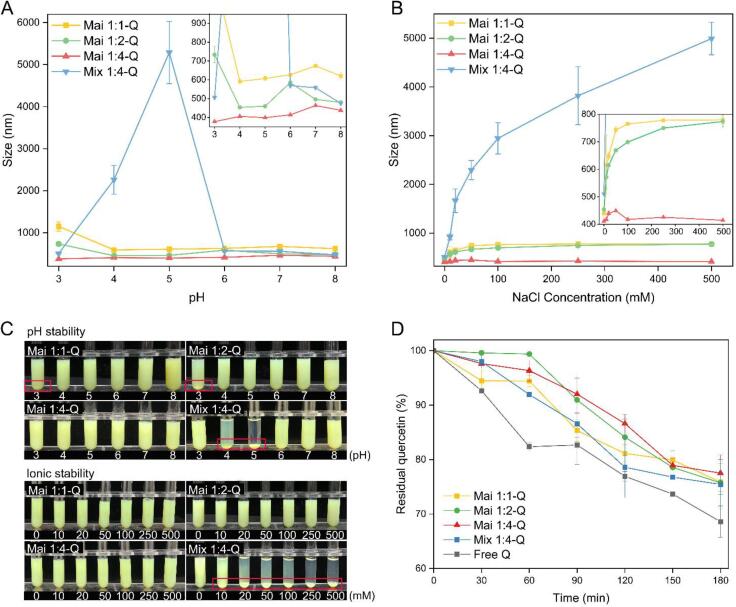
Effect of (A) pH and (B) NaCl concentration on particle size and (C) appearance of Mai 1:1-Q, Mai 1:2-Q, Mai 1:4-Q, Mix 1: 4-Q. (D) The photochemical stability of quercetin under UV-light irradiation.

As shown in Fig. 3A and C, Mix1:4-Q exhibited extensive precipitation at pH 4 and 5, which are near the isoelectric point of ALA (pH 4.2 and 4.5). Differently, all three glycated ALA stabilized micelles had the smallest particle size at pH 4 and there was no significant change from pH 4 to 8. Mai 1:4-Q had the best stability across the entire pH range, showing smallest average sizes maintained between 377.80 nm and 468.67 nm. This indicates that the non-covalently reacted dextran was unable to prevent ALA aggregation caused by lack of electrostatic repulsion, while the dextran covalently grafted on ALA could provide strong steric repulsion to maintain the stability of the micelles.

#### Ionic stability

The effect of ion strength on micelles stability was performed in Fig. 3B and C. There was a dramatic increase in the mean particle size of Mix 1:4 when the salt level was higher than 10 mM, due to the rise of ionic strength shielded the electrostatic repulsion between proteins. By contrast, micelles constructed with glycated ALA all showed the superior salt tolerance, especially Mai 1:4-Q, whose particle size remained relatively stable between 410.8 and 449.07 nm even in 500 mM NaCl solution. As for Mai 1:1-Q and Mai 1:2-Q, when NaCl concentration increased to 50 mM, their particle size also showed a steep trend of rise from 439.5 nm to 743 nm and 453.9 nm to 669 nm, respectively, which then were gradually stabilized until NaCl concentration reach 100 mM. This trend was the result of the balance between the electrostatic screening effect of NaCl induced protein aggregation and the steric hindrance effect of covalently grafted dextran.

#### Photothermal stability

It had been reported that quercetin would undergo decomposition as the result of C-ring opening under UV light irradiation (Zvezdanović, Stanojević, Marković, & Cvetkovic, 2012). From Fig. 3D, only 68.61 ± 2.87% of free quercetin remained when exposed to UV light for 180 min. After encapsulation, the retention rates of quercetin were enhanced to 75.88 ± 3.41%, 75.74 ± 2.11%, 77.52 ± 3.32% and 75.45 ± 4.56% by Mai 1:1-Q, Mai 1:2-Q, Mai 1:4 and Mix 1:4-Q, respectively. Compared to the free quercetin, the four ALA-dextran based encapsulation systems were able to effectively protect quercetin from UV light caused degradation (p < 0.05), but there was no significant difference among themselves (p > 0.05).

### Digestive fate of quercetin-loaded micelles

#### In vitro release properties

**Fig. 4:**
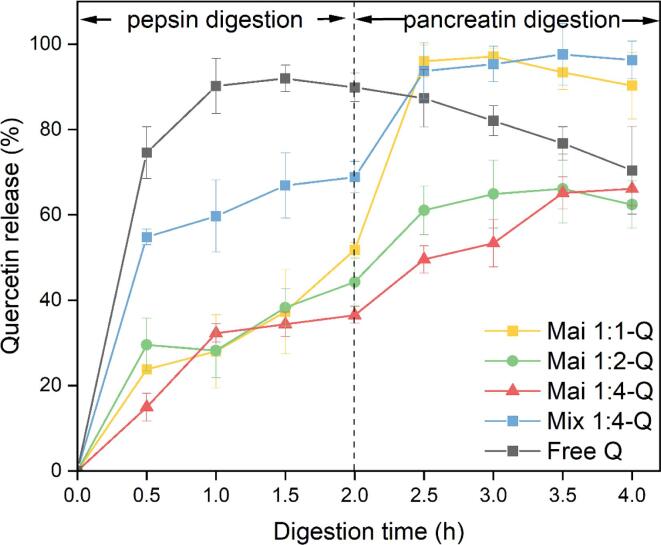
Quercetin release profile from Mai 1:1-Q, Mai 1:2-Q, Mai 1:4-Q and Mix 1: 4-Q.

The release characteristics of quercetin during the simulated digestion process was depicted in Fig. 4. Free quercetin was released 90.18% after gastric digestion within 1 h, and subsequently performed a rapid degradation in the SIF. At the end of digestion, there was only 70.42% free quercetin left, caused by poor stability of quercetin if pH was higher than 7 (Moon, Wang, Dicenzo, & Morris, 2008). The second rapid release was found in Mix 1:4-Q, which experienced burst release within 30 min in both SGF (from 0 to 54.79%) and SIF (from 68.85% to 93.71%). However, there was no obvious degradation of quercetin from Mix 1:4-Q in the intestinal phase, suggesting that the physical mixture-based delivery system still could provide protection to quercetin. All the ALA-dextran conjugates stabilized micelles exhibited the sustained release process in SGF. At the end of gastric digestion, the amount of quercetin released by Mai 1:1-Q, Mai 1:2-Q and Mai 1:4-Q are 51.78%, 44.27% and 36.52%, respectively. In the intestine phase, Mai 1:2-Q and Mai 1:4-Q still performed a slow-release trend, releasing 62.41% and 66.15% of quercetin at the end of digestion, while 96.03% of quercetin were released in Mai 1:1-Q after exposure to SIF for 30 min.

#### Hydrolysates analysis by SDS-PAGE

**Fig. 5:**
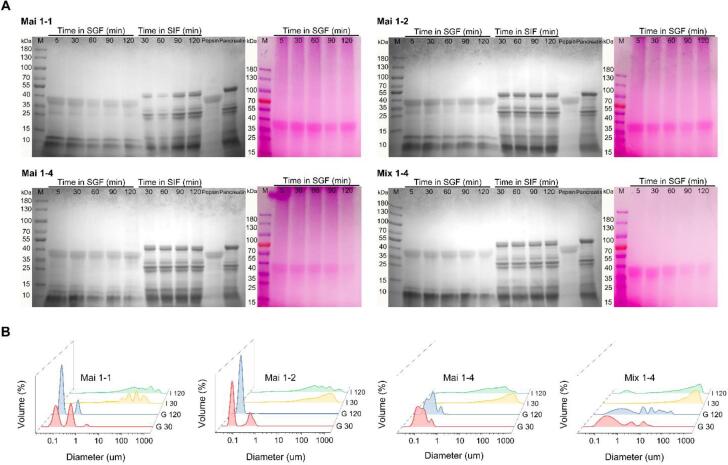
(A) SDS-PAGE analysis and (B) particle distribution of Mai 1:1-Q, Mai 1:2-Q, Mai 1:4-Q, Mix 1: 4-Q following *in vitro* digestion.

In Fig. 5A, the evolution of the protein hydrolysis in quercetin-loaded micelles was monitored by SDS-PAGE. In Mix 1:4-Q, the bands of native ALA became blurred within 5 min of gastric digestion, and the bands almost disappeared after 2 h. It indicates dextran was unable to prevent protein digestion by only relying on noncovalent interactions. As for the glycated ALA-based micelles, combining the results of protein and carbohydrate staining gels, ALA-dextran conjugates were existed throughout the entire gastric phase, and there was no significant change in molecular weight distribution and color depth after 2 h. However, the conjugates in all three micelles were no longer visible in SIF, and the peptic fragments less than 10 kDa were increased, suggesting that all the ALA were hydrolyzed into small peptides. The resistance of ALA-dextran conjugates to hydrolysis was attributed to the steric hindrance provided covalently linked dextran, which interfered the contact between proteases and protein (Li, Ye, & Singh, 2021). The result of electrophoresis analysis is consistent with the release profile of quercetin during the digestion process. The rapid hydrolysis of ALA in Mix 1:4-Q by pepsin leaded to the destruction of the delivery system and the burst release of quercetin. The sustained release of quercetin in all three glycated ALA-based micelles was due to the protection from conjugated dextran. Nevertheless, the depletion of the conjugates in the intestinal stage only caused the burst release of Mai 1:1-Q, but did not obviously accelerate the release of Mai 1:2-Q and Mai 1:4-Q. This difference probably because the latter two micelles had the GD of dextran that cannot be decomposed by digestive enzymes, and the higher EE than Mai 1:1-Q, and accordingly delayed the release of quercetin in SIF.

#### Particle size distribution

As observed in Fig. 5B, the size distribution of Mix 1:4-Q became significantly broadened and heavy flocculation occurred during the gastric digestion. After incubation in SGF for 30 min, the particle size of Mai 1:1-Q, Mai 1:2-Q and Mai 1:4-Q presented a bimodal distribution: one peak was below 100 nm and the other was around 350 nm. Compared to the initial particle sizes (around 400–500 nm), the decrease in particle size reflected the occurrence of partial hydrolysis of glycated ALA. At the end of gastric digestion, both the size distribution of Mai 1:1-Q and Mai 1:2-Q became dominated by the peak below 100 nm, while the size distribution of Mai 1:4-Q did not change significantly. During the intestinal stage, all of the delivery systems exhibited similar aggregation and had large mean diameters (≈750 nm), might be attributed to the released quercetin and the effect of bile salts. The destabilization of protein-based delivery systems under simulated digestion may occur for some reasons, including loss of charge due to pH changes, electrostatic screening due to an increased ionic strength, and proteolysis by pepsin (Davidov-Pardo, Pérez-Ciordia, MaríN-Arroyo, & Mcclements, 2015). Mai 1:4-Q had the best stability during the gastric digestion, which was consistent with its best pH stability, ionic strength stability, resistance to pepsin hydrolysis, and the ability to sustained release of quercetin.

## Conclusion

ALA-dextran conjugates were successfully fabricated by Maillard reaction and used to form micelles to encapsulate water-insoluble quercetin. The micelles Mai 1:4-Q had the highest EE and showed the best pH, ionic strength stability, and the UV irradiation protection of quercetin. Through the steric hindrance provided by covalently linked dextran, ALA-dextran conjugates exhibited resistance to the hydrolysis of enzymes, and realized the sustained release of quercetin. Remarkably, the comparison of Mai 1:4-Q and Mix 1:4-Q implied that covalent protein-polysaccharide conjugates could provide superior protection for bioactive compounds than non-covalent protein-polysaccharide mixtures. Appropriate degree of grafting may be a key factor in determining the EE, stability and digestion resistance of the conjugates-stabilized delivery systems. These conclusions demonstrate the great potential of glycated proteins for oral delivery. Further studies could focus on the peptide and amino acids that released from the ALA-dextran conjugates during simulated digestion. Moreover, *in vivo* digestion and the oral pharmacokinetics study of quercetin-loaded micelles stabilized by ALA-dextran conjugates were also worth to be investigated.

## Declaration of Competing Interest

The authors declare that they have no known competing financial interests or personal relationships that could have appeared to influence the work reported in this paper.
