# Supplementary material for: Glycated α-lactalbumin based micelles for quercetin delivery: Physicochemical stability and fate of simulated digestion
Source: Food Chem X. 2022 Feb 15;13:100257. doi: 10.1016/j.fochx.2022.100257 (PMC9039997; doi:10.1016/j.fochx.2022.100257)
Supplement: Supplementary data 1 [file mmc1.docx]

**Glycated α-lactalbumin based micelles for quercetin delivery: Physicochemical stability and Fate of simulated digestion**

Wanting Yin ^a^, Luqing Song ^a^, Yanan Huang ^a^, Fang Chen ^a^, Xiaosong Hu ^ab^, Lingjun Ma ^ab, *^, Junfu Ji ^ab, *^

^a^ College of Food Science and Nutritional Engineering, National Engineering Research Center for Fruit and Vegetable Processing, China Agricultural University, Key Lab of Fruit and Vegetable Processing, Ministry of Agriculture and Rural Affairs, Beijing 100083, China

^b^ Xinghua Industrial Research Centre for Food Science and Human Health, China Agricultural University, Xinghua, 225700, China

^*^Corresponding author.

Tel: +86-10-62737434; Fax: +86-10-62737645-18

E-mail address: [junfu.ji@cau.edu.cn](mailto:junfu.ji@cau.edu.cn)


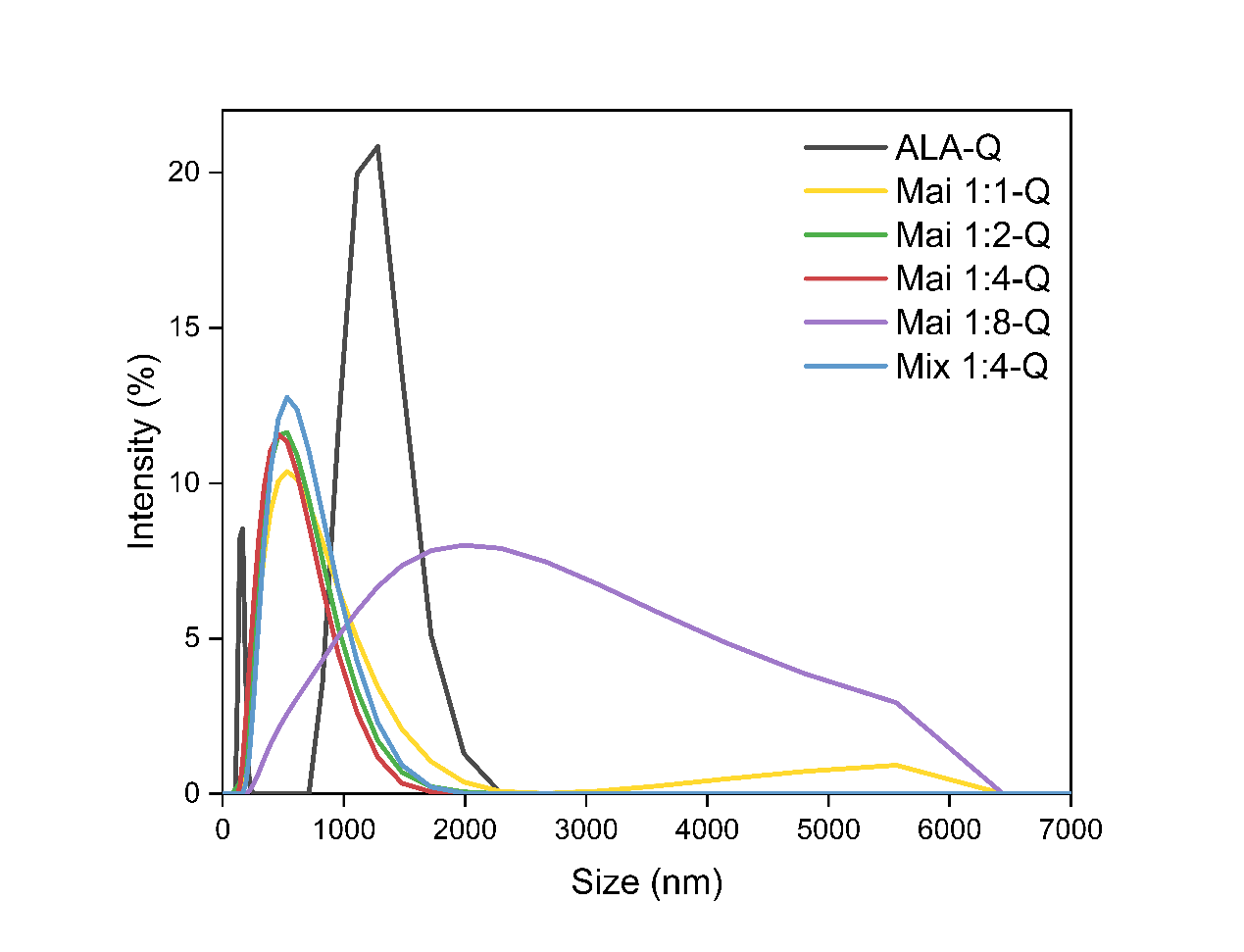


**Figure S1**. Size distribution of ALA-Q, Mai 1:1-Q, Mai 1:2-Q, Mai 1:4-Q, Mai 1:8-Q and Mix 1:4-Q.
